# Job vacancies at the European Centre for Disease Prevention and Control (ECDC)

**DOI:** 10.2807/1560-7917.ES.2023.28.49.2312076

**Published:** 2023-12-07

**Authors:** Eurosurveillance editorial team

**Affiliations:** 1 European Centre for Disease Prevention and Control (ECDC), Stockholm, Sweden

The European Centre for Disease Prevention and Control (ECDC) invites applications for the following positions:

 

**Chief Scientist / Head of Unit Scientific Methods and Standards**
The application deadline expires on 9 Jan 2024.

  

**Head of Unit Disease Programmes**
The application deadline expires on 9 Jan 2024.

  

**Data Scientist**
The application deadline expires on 9 Jan 2024.

  

**Scientific Officer Emergency Preparedness and Response**
The application deadline expires on 16 Jan 2024.

  

**Principal Expert Public Health Training**
The application deadline expires on 16 Jan 2024.

 

For more information, please visit the ECDC website: https://erecruitment.ecdc.europa.eu/?page=advertisement

